# Supplementary material for: Acoustic and perceptual impact of face masks on speech: A scoping review
Source: PLoS One. 2023 Aug 25;18(8):e0285009. doi: 10.1371/journal.pone.0285009 (PMC10456191; doi:10.1371/journal.pone.0285009)
Supplement: S1 Appendix — (DOCX) [file pone.0285009.s001.docx]

Full search terminology for scoping review

| **Search category** | **PubMed** | **Embase** | **PsycINFO** | **Web of Science** | **Google Scholar** |
| --- | --- | --- | --- | --- | --- |
| **[Terms to identify acoustic and perceptual outcomes] AND** | ("speech"[Title/Abstract] OR "speech"[MeSH Terms]  OR "intelligib*"[Title/Abstract] OR "intelligib*"[MeSH Terms]  OR "speech perception"[Title/Abstract] OR "speech perception"[MeSH Terms]  OR "acoustic*"[Title/Abstract] OR "acoustic*"[MeSH Terms]) | ('speech intelligibility'/exp OR 'speech perception'/exp OR 'speech'/exp  OR 'speech intelligibility':ab,ti OR 'speech perception':ab,ti OR 'speech':ab,ti OR 'acoustic' OR 'acoustic':ab,ti) | (TI ("intelligib*" OR "speech perception" OR "acoustic*" OR “speech”)  OR AB ("intelligib*" OR "speech perception" OR "acoustic*" OR “speech”)  OR DE ("intelligib*" OR "speech perception" OR "acoustic*" OR “speech” OR “oral communication”)) | (TI=("intelligib*" OR "speech perception" OR "acoustic*")  OR AB=("intelligib*" OR "speech perception" OR "acoustic*")  OR SU=("intelligib*" OR "speech perception" OR "acoustic*")) | (intelligib* OR “speech perception” OR acoustic*) |
| **[Terms to identify relevant face coverings] AND** | ("face mask*"[Title/Abstract] OR "face mask*"[MeSH Terms]  OR "face-mask*"[Title/Abstract] OR "face-mask*"[MeSH Terms]  OR "surgical mask*"[Title/Abstract] OR "surgical mask*"[MeSH Terms]  OR "facepiece*"[Title/Abstract] OR "facepiece*"[MeSH Terms]   OR "respiratory protect*"[MeSH Terms] OR "respiratory protect*"[Title/Abstract]  OR "respirator*"[Title/Abstract] OR “respirator”[MeSH Terms]  OR "N95*"[Title/Abstract] OR "N95"[MeSH Terms]  OR "face shield*"[Title/Abstract] OR "face shield*"[MeSH Terms]  OR "face cover*"[Title/Abstract] OR "face cover*"[Title/Abstract]  OR "face protect*"[Title/Abstract] OR "face protect*"[MeSH Terms]  OR “personal protective equipment”[Title/Abstract]  OR “personal protective equipment”[MeSH Terms]) | ('face mask'/exp OR 'face mask ventilation'/exp OR 'surgical mask'/exp OR 'ventilator'/exp OR 'n95'/exp OR 'minimally 94 percent efficient filtering facepiece respirator'/exp OR 'face shield'/exp OR 'protective equipment'/exp  OR 'face mask':ab,ti OR 'face mask ventilation':ab,ti OR 'surgical mask':ab,ti OR 'ventilator':ab,ti OR 'n95':ab,ti OR 'minimally 94 percent efficient filtering facepiece respirator':ab,ti OR 'face shield':ab,ti OR 'protective equipment':ab,ti) | (TI ( "mask" OR "respirator" OR "surgical mask" OR "respiratory protect*" OR "N95" OR "face shield" OR "fac* cover*" OR "mask cover*" OR "fac* protect*" )  OR AB ( "mask" OR "respirator" OR "surgical mask" OR "respiratory protect*" OR "N95" OR "face shield" OR "fac* cover*" OR "mask cover*" OR "fac* protect*" )  OR DE personal protective equipment OR DE ( "mask" OR "respirator" OR "surgical mask" OR "respiratory protect*" OR "N95" OR "face shield" OR "fac* cover*" OR "mask cover*" OR "fac* protect*" OR "personal protective equipment" )) | (TI=("face mask*"  OR "surgical mask*"  OR "facepiece*"   OR "respiratory protect*"  OR "N95*"  OR "face shield*"  OR "face cover*"  OR "face protect*"  OR “personal protective equipment”  OR “respirator”)  OR  AB=("face mask*"  OR "surgical mask*"  OR "facepiece*"   OR "respiratory protect*"  OR "respirator*”  OR "N95*"  OR "face shield*"  OR "face cover*"  OR "face protect*"  OR “personal protective equipment”)  OR  SU=("face mask*"  OR "surgical mask*"  OR "facepiece*"   OR "respiratory protect*"  OR "N95*"  OR "face shield*"  OR "face cover*"  OR "face protect*"  OR “personal protective equipment”)) | ("face mask" OR facemask* OR "surgical mask"  OR respirator OR “n95”  OR “face shield” OR “face cover”  OR "face covering" OR “facepiece") |
| **[Terms to identify studies related to speech and or voice]** | ("speech"[MeSH Terms] OR "speech"[Title/Abstract]  OR “voice”[MeSH Terms] OR “voice”[Title/Abstract]) | ('speech'/exp OR 'voice'/exp  OR 'speech':ab,ti OR 'voice':ab,ti) | (TI (“speech” OR “voice”)  OR AB (“speech” OR “voice”)  OR DE (“speech” OR “voice” OR “oral communication”)) | (TI=(speech OR voice)  OR AB=(speech OR voice)  OR SU=(speech OR voice)) | (speech OR voice) |
